# Alpha-1-Antitrypsin: A Novel Human High Temperature Requirement Protease A1 (HTRA1) Substrate in Human Placental Tissue

**DOI:** 10.1371/journal.pone.0109483

**Published:** 2014-10-20

**Authors:** Violette Frochaux, Diana Hildebrand, Anja Talke, Michael W. Linscheid, Hartmut Schlüter

**Affiliations:** 1 Department of Chemistry, Humboldt-Universität zu Berlin, Berlin, Germany; 2 Department of Clinical Chemistry, University Medical Center Hamburg-Eppendorf, Hamburg, Germany; 3 ProteaImmun GmbH, Berlin, Germany; Imperial College London, United Kingdom

## Abstract

The human serine protease high temperature requirement A1 (HTRA1) is highly expressed in the placental tissue, especially in the last trimester of gestation. This suggests that HTRA1 is involved in placental formation and function. With the aim of a better understanding of the role of HTRA1 in the placenta, candidate substrates were screened in a placenta protein extract using a gel-based mass spectrometric approach. Protease inhibitor alpha-1-antitrypsin, actin cytoplasmic 1, tropomyosin beta chain and ten further proteins were identified as candidate substrates of HTRA1. Among the identified candidate substrates, alpha-1-antitrypsin (A1AT) was considered to be of particular interest because of its important role as protease inhibitor. For investigation of alpha-1-antitrypsin as substrate of HTRA1 synthetic peptides covering parts of the sequence of alpha-1-antitrypsin were incubated with HTRA1. By mass spectrometry a specific cleavage site was identified after met-382 (AIPM^382^↓^383^SIPP) within the reactive centre loop of alpha-1-antitrypsin, resulting in a C-terminal peptide comprising 36 amino acids. Proteolytic removal of this peptide from alpha-1-antitrypsin results in a loss of its inhibitor function. Beside placental alpha-1-antitrypsin the circulating form in human plasma was also significantly degraded by HTRA1. Taken together, our data suggest a link between the candidate substrates alpha-1-antitrypsin and the function of HTRA1 in the placenta in the syncytiotrophoblast, the cell layer attending to maternal blood in the villous tree of the human placenta. Data deposition: Mass spectrometry (MS) data have been deposited to the ProteomeXchange with identifier PXD000473.

## Introduction

Human HTRA1 belongs to the HtrA (high temperature requirement A) serine protease family. HtrA was first identified in Escherichia coli (E. coli) and is essential for the bacterial survival at high temperature [1]. The bacterial protease acts as a chaperone and degrades misfolded proteins at elevated temperatures [2]. Four human homologues of HtrA have been identified to date: HTRA1, HTRA2, HTRA3 and HTRA4. The human protease HTRA1 was first isolated from SV40-transformed fibroblast where it has been identified as a down-regulated gene [3]. HTRA1 contains a trypsin-like serine protease domain, a PDZ domain, an insulin-like growth factor binding protease (IGFBP) domain and a Kazal-type inhibitor domain [3]. Full-length HTRA1 has a molecular weight of ∼50 kDa. Several ∼30 kDa additional protein species of HTRA1 are known [4]. After binding a substrate, HTRA1 switches to an active conformation. In the active conformation, the catalytic triade is properly positioned for catalysis of the proteolytic reaction. Crystal structure of HTRA1 shows that HTRA1 crystallises as a trimer [5]. This oligomerization may play a role in proteolytic activity of the protease, as observed by E. coli HTRA [6].

Though its exact role is not well understood, HTRA1 seems to be involved in several pathologies, as rheumatoid arthritis [7], osteoarthritis [4], Alzheimer’s disease [8], age-related macular degeneration [9] [10] [11] and some types of cancer [12] [13]. In ovarian cancer loss of HTRA1 expression may contribute to malignant phenotype whereas overexpression inhibits proliferation and cell growth.

Recently, several substrates of HTRA1 were identified and different hypotheses of its biological function were published. The tumour suppressor property of HTRA1 was correlated with its association to microtubule in cancer cells, which influences the cell motility [14], [15]. Grau et al. demonstrated that HTRA1 degrades fibronectin which seems to lead to increasing mRNA and protein amounts of different matrix metallopeptidases (MMPs) in human synovial fibroblasts and finally to the degradation of the cartilage [7]. The same group suggests that HTRA1 is implicated in Alzheimeŕs disease by degradation of amyloid β in the human brain, while the application of an HTRA1 inhibitor causes accumulation of amyloid β in astrocyte cells [8]. In the same way, Tennstaedt et al. suggest a protein quality control function for HTRA1 through degradation of aggregated tau [16]. HTRA1 was reported to have apoptotic properties. He et al. identified the substrate XIAP, a member of the “inhibitor of apoptosis family” (IAP) and described the contribution of HTRA1 to chemoresistance [17]. The influence on apoptotic process may correlate with the tumour suppressor properties of the protease [15].

HTRA1 gene is expressed and translated in different tissues and organs. The highest levels of HTRA1 were found in the placenta [18]. Using in situ hybridization and immunohistochemistry methods the level of HTRA1 was found to be especially high in the third trimester of gestation [19]. Furthermore the level of HTRA1 in placenta with preeclampsia (PE) or trophoblastic diseases was reported to be deregulated compared with normal placenta [20]–[22].

To understand the functional roles of a protease, it is essential to elucidate its substrate repertoire [23]. As a proof that a defined protein is a substrate of a given protease, the purified candidate substrate should be proteolysed in the presence of the target protease. Using peptide libraries substrate specificities of proteases of interest can be determined. However, for the identification of a native substrate of a target protease the biological context is crucial. Many parameters contribute to the role of the protease in vivo, as tertiary and quaternary structure of the substrate or the cooperation of co-factors and ligands.

In this work, we have chosen a cell-free gel-based mass spectrometric approach to investigate the substrate repertoire of HTRA1 in the human placenta. The degradomics approach we used is fast and allows the identification of candidate substrates in the complex mixture of protein extracts from tissues. With this method several new candidate substrates of HTRA1 were identified, giving an insight into the biological function of the protease HTRA1 in the placenta.

## Materials and Methods

### Ethics Statement

In this work we have used the placentas of healthy women, who had given their written consent.

The use of this material has been approved by the ethics committee Ärztekammer Hamburg.

### Protease and substrates

Recombinant human HTRA1 was expressed in insect cells and purified from insect cell culture supernatants (ProteaImmun GmbH, Berlin, Germany). Preparation was analysed by SDS-PAGE using coomassie blue staining (≥70% of total protein). Protein bands were cut, digested in-gel with trypsin and analyzed via Mass spectrometry to confirm identity of the protease and to identify the impurities. All protein bands were found to be HTRA1 or fragments of HTRA1. A1AT was purified from pooled human plasma (ProteaImmun GmbH, Berlin, Germany), and purity was confirmed by SDS-PAGE using coomassie blue staining (≥95% of total protein). β-Casein was purified from bovine Milk (Sigma), and purity was confirmed by SDS-PAGE using coomassie blue staining (≥98% of total protein).

### Preparation of a placenta protein extract from the placenta

Fresh placentas of healthy women, who had given their written consent, were cut into small pieces (at 4°C) and immediately frozen in liquid nitrogen. The frozen material was lyophilised, pulverised and homogenised by Ultra-Turrax at 4°C (IKA-Werke GmbH, Staufen) in phosphate buffered saline (PBS; 137 mM NaCl, 26 mM KCl, 14 mM KH_2_PO_4_, 80 mM Na_2_HPO_4_, pH 7.4; 4°C). After centrifugation (15,000 g, 4°C, 30 min) the supernatant was filtered by a 0.45 µm filter (Millipore Millex-HV Hydrophilic PVDF filter) followed by a second filtration using an Amicon Ultra-15 filter with a 15 kDa membrane. The total protein concentration of the resulting supernatant was determined using a Bradford assay [24].

### Enrichment of HTRA1 using immunobeads, western blot of the placenta proteins

50 mg/ml placenta supernatant was diluted to a final concentration of 15 mg in 3 ml PBS. The placenta proteins were incubated with 10 µl anti-HTRA1-beads (ProteaDetect Extract HtrA1ProteaImmun GmbH, Berlin, Germany) for 3 hours at 37°C. After several washing steps the beads were incubated for 7 min at 95°C after addition of sample buffer without dithiothreitol (DTT) to elute the enriched HTRA1 protease. The eluent was loaded on a sodium dodecyl sulphate (SDS) gel. HtrA1 was detected by Western blot using the same monoclonal HTRA1 antibody as used in the anti-HTRA1-beads (ProteaImmun GmbH, Berlin, Germany).

### Incubation of β-casein with HTRA1 in the Presence or absence of different protease-inhibitors followed by 1D-gel electrophoresis or HPLC analysis

A range of protease inhibitors (EDTA ethylenediaminetetraacetic acid, E64 [N-(trans-epoxysuccinyl)-L-leucine 4 guanidinobutylamide], pepstatinA, bestatin, leupeptin, AEBSF [4-(2-aminoethyl)benzenesulfonyl fluoride hydrochloride], and aprotinin, Sigma) were added separately to an incubation mixture containing 5 µg β-casein in 5 µl buffer (150 mM NaCl, 5 mM CaCl_2_, 50 mM Tris-HCl, pH 7.4) and 0.5 µg recombinant human HTRA1 expressed in insect cells and purified from insect cell culture supernatants (0.2 µg/µl in protease buffer: 150 mM NaCl, 5 mM KCl, 50 mM imidazol, 50 mM Tris-HCl, 0.05% Brij-35, pH 7.5, ProteaImmun GmbH, Berlin, Germany) and incubated at 37°C. Aliquots from the incubation mixture were taken at different times and separated by SDS-Page as described by Laemmli [25] using 15% acrylamide final concentration. After electrophoresis the gels were stained with Coomassie blue. On the SDS-polyacrylamide gel electrophoresis (SDS-PAGE), proteolytic activity was registered as present, when no full-length β-casein was detected after 1 hour incubation. For validation, this experiment was repeated using high-performance liquid chromatography (HPLC) with UV detection for the analysis of the reaction products. Aliquots of the incubation mixture (150 pmol of the β-Casein) were injected on a reversed-phase (RP) column (Luna 00A-4041-C18; 5 µm, 150×0.5 mm; Phenomenex) driven by the HPLC system (Agilent 1200 system; Agilent Technologies, Waldbronn, Germany). Separation was performed using a binary mobile phase (eluent A: 94.9% deionized water, 5% acetonitrile, 0.1% formic acid (v/v/v); eluent B: 99.9% acetonitrile, 0.1% (v/v) formic acid) with a flow rate of 50 µl min^−1^. Peptides were separated using a 60 min gradient as follow: 0–35 min 5% to 70% B; 35–37 min 70% B; 37–40 min 5% B; 40–60 min 70–5% B. MS detection was used to verify the identity of the β-casein peak of the UV-chromatogram. HPLC system was coupled with a Fourier transform ion cyclotron resonance mass spectrometer (FT-ICR-MS) (Finnigan LTQ FT ULTRA Thermo Fisher Scientific, Bremen, Germany). Electrospray ionization source (ESI) of the FT-ICR was working at 5.0 kV. Positive ionisation and a transfer capillary temperature of 275°C were applied.

### Incubation of the placenta proteins with HTRA1

225 µg of the placenta proteins were incubated with 25 µg purified recombinant human HTRA1 expressed in insect cells in a final volume of 285 µl for 4 hours at 37°C (+HTRA1). As a control 225 µg of the extracted placenta proteins were incubated with the same volume of protease buffer without HTRA1 using the same incubation conditions (−HTRA1). After incubation 2 ml cold acetone (−20°C) was added to each sample. After storing 1 hour at −20°C the precipitates were collected by centrifugation (20 min, 4°C, 15.000 g) and separated from the liquid phase. The pellets were washed 2 times with 90% acetone, 10% water at −20°C and centrifuged under same conditions. The pellets were finally dissolved in a sample buffer (9 M urea, 70 mM DTT, 2% ampholytes 2–4) for isoelectric focusing. For validation, incubation experiment analysed by 2-DE separation was performed in two independent replicates.

For further validation this experiment was repeated with addition of an inhibitor cocktail (P8340, sigma, concentrations of the inhibitors in the incubation solution are: AEBSF 1.04 mM, aprotinin 0.80 µM, bestatin 40 µM, E-64 14 µM, leupeptin 20 µM, pepstatin A 15 µM), or with addition of the protease inhibitor tissue inhibitor of metalloproteinase-1 (Timp-1, Sigma, ratio 10∶1 total protein to inhibitor weight).

### 2-D-gel electrophoresis

The incubated samples (+HTRA1/−HTRA1) were subjected to 2D gel electrophoresis analysis (2-DE). Two dimensional gel electrophoresis was performed according to Proteome Factory's 2-D electrophoresis technique based on Klose and Kobalz [26]. 250 µg of total protein was applied to vertical rod gels (9 M urea, 4% acrylamide, 0.3% piperazine diacrylamide, 5% glycerol, 0.06% tetramethylethylenediamine (TEMED) and 2% carrier ampholytes (pH 2–11)) for isoelectric focusing (IEF) at 8820 Vh in the first dimension. After focusing, the IEF gels were incubated in equilibration buffer, containing 125 mM Trisphosphate (pH 6.8), 40% glycerol, 65 mM DTT, and 3% SDS for 10 minutes and subsequently frozen at −80°C. The second dimension SDS-PAGE gels (23×30×0.1 cm) were polymerized from 375 mM Tris-HCl buffer (pH 8.8), 15% arcylamide, 0.2% bisarcylamide, 0.1% SDS and 0.03% TEMED. After thawing, the equilibrated IEF gels were immediately applied to SDS-PAGE gels. Electrophoresis was performed using a two-step increase of current, starting with 15 min at 65 mA, followed by a run of 6 h at 140 mA, until the front reached the end of the gel. After electrophoresis, the analytical gels were stained with MS compatible silver nitrate (FireSilver staining kit PS-2001, Proteome Factory AG, Berlin, Germany). For image analysis the 2-DE gels used for comparison analysis were digitized at a resolution of 150 dpi using a PowerLook 2100XL with transparency adapter. Two-dimensional image analysis was performed using the Proteomweaver software (Definiens AG, Munich, Germany). For subsequent analysis selected protein spots were digested in-gel.

### Trypsin in-gel digestion

For in-gel digestion selected protein spots were cut out, transferred to vials, and washed three times using 100–200 µl buffer (50% acetonitrile, and 50% 25 mM ammonium hydrogen carbonate (v/v)). Then the gel pieces were dehydrated using 50 µl acetonitrile. The supernatant was removed and the gel pieces were dried for 20 min at 37°C. 20 to 30 µl of modified trypsin (0.01 g/l, Promega) in 25 mM ammonium hydrogen carbonate was added subsequently and after 30 min 20 µl of 25 mM ammonium hydrogen carbonate buffer was added to each vial; trypsin digestion was carried out for 12 h at 37°C. After centrifugation, the supernatant was carefully removed, and the peptides were extracted twice from the gel by adding 50 µl of the following extraction solution: 50% acetonitrile, 50% formic acid (5%). Finally all pellets were extracted with 50 µl of acetonitrile. The supernatants were pooled, evaporated to dryness by a vacuum concentrator and redissolved in 20 µl 0.1% formic acid (v/v) for mass spectrometric analysis.

### Mass spectrometry-based proteomic analysis

For nanoHPLC/ESI-MS/MS, a 1100 HPLC system (Agilent) was used. Separation was performed on a Zorbax 300 SB-C18 (150 mm×75 µm) column with a Zorbax 300 SB-C18 (0.3 mm×5 mm; Agilent Technologies; Waldbronn, Germany) enrichment column and a binary mobile phase water/acetonitrile gradient with a maximum flow rate of 0.30 µl min^−1^. Peptides were separated using a 35 min gradient. The eluents used were as follows: A-94.9% deionized water, 5% acetonitrile, 0.1% formic acid (v/v/v); B­99.9% acetonitrile, 0.1% (v/v) formic acid. The separation column was coupled to a Finnigan LTQ FT ULTRA mass spectrometer (Thermo Fisher Scientific, Bremen, Germany) using a nanomate ESI interface (Advion) working at 1.7 kV. Positive ionisation and a transfer capillary temperature of 200°C were applied. Mass spectrometric detection was performed by a data-dependant method of acquisition controlled by Xcalibur 2.07 software version (Thermo Scientific, Bremen) where the five most intense precursor ions detected in the full MS scan (FT-ICR) were selected and fragmented in the Ion Trap by collision induced dissociation (CID) (35% energy, 4 amu mass isolation width). Proteome Discoverer 1.3 and the search engine SEQUEST (Thermo Scientific, Bremen) were used for data analysis. The MS/MS raw data were directly analysed with the Proteome Discoverer software using the following spectrum selector settings; minimum precursor mass 350 Da, maximum precursor mass 5000, total intensity threshold 100, minimum peak count 10, signal-to-noise threshold 5 (FT-only). The identification searching parameters were: tryptic digestion, 2 missed cleavages, deamidated (N), oxidation (M) and propionamide (C) dynamic modifications, precursor mass tolerance 3 ppm and fragment mass tolerance 0.5 Da. The database UniProtKB/Swiss-Prot homo sapiens (September 2013, 20′267 Proteins) was chosen for protein identification. A list of contaminants was removed from this database, namely keratin, type I and type II cytoskeletal and trypsin. A reverse database was used to prevent false positive identification. As discrimination criteria, protein identifications containing less than 2 peptides and SEQUEST scores lower than 40 were discarded. The mass spectrometry proteomics data included database and list of the contaminants have been deposited to the ProteomeXchange Consortium (http://proteomecentral.proteomexchange.org) via the PRIDE partner repository with the dataset identifier PXD000473 [27].

### In vitro cleavage of pure plasma A1AT with HTRA1

10 µg A1AT purified from pooled human plasma (ProteaImmun GmbH, Berlin, Germany) solved in 10 µl PBS (137 mM NaCl, 26 mM KCl, 14 mM KH_2_PO_4_, 80 mM Na_2_HPO_4_, pH 7.4) was incubated with 0.5 µg HTRA1 (0.2 g/l) at 37°C over night (17 h). An aliquot was taken at different times. The incubation experiment was repeated three times and subjected to 2-D electrophoresis analysis as described above or to SDS-PAGE analysis (10% acrylamide final concentration). The gel was stained after electrophoresis with Coomassie blue. Selected protein spots were digested in-gel. The obtained peptides were analysed by LC-MS.

### Incubation of A1AT with HTRA1 and β-casein

20 µg A1AT (1 g/l in PBS) was incubated with 0.4 µg HTRA1 (0.2 g/l) and 20 µg β-Casein (0.1 g/l in PBS) at 37°C. Aliquots were taken at time 0 h, 30 min, 1 h and 3 h. The same experiment was repeated without A1AT, as positive control.

The aliquots were analysed by SDS Page using 15% acrylamide final concentration. The gel was stained after electrophoresis with Coomassie blue.

### Incubation of A1AT peptides with HTRA1

5 µl of a 100 µM solution of the synthetic peptides A1AT(361–384), A1AT(379–400) and A1AT(396–418) (AG Kloetzel, Charité, Berlin) were incubated each separately with 12.5 µl HTRA1 (0.2 g/l) in a molar ratio 10∶1 substrate to protease at 37°C. The same incubation experiment was performed without addition of HTRA1 as negative control. Aliquots from the incubation mixture were taken at different times and after dilution to 2 µM with 50% methanol, 50% formic acid (0.1%) analysed by matrix-assisted laser desorption/ionization (MALDI) Orbitrap MS (MALDI-Orbitrap-XL, Thermo Fisher Scientific, Bremen, Germany). A 3.5 mg/ml alpha-cyano-4-hydroxycinnamic acid solution in 50% methanol, 50% formic acid (0.1%) was used as matrix. The fragments were identified by molecular ions with an accuracy of 1–4 ppm and a laser energy of 8 µJ.

## Results

### Only full-length HTRA1-form in the placenta

**Figure 1:**
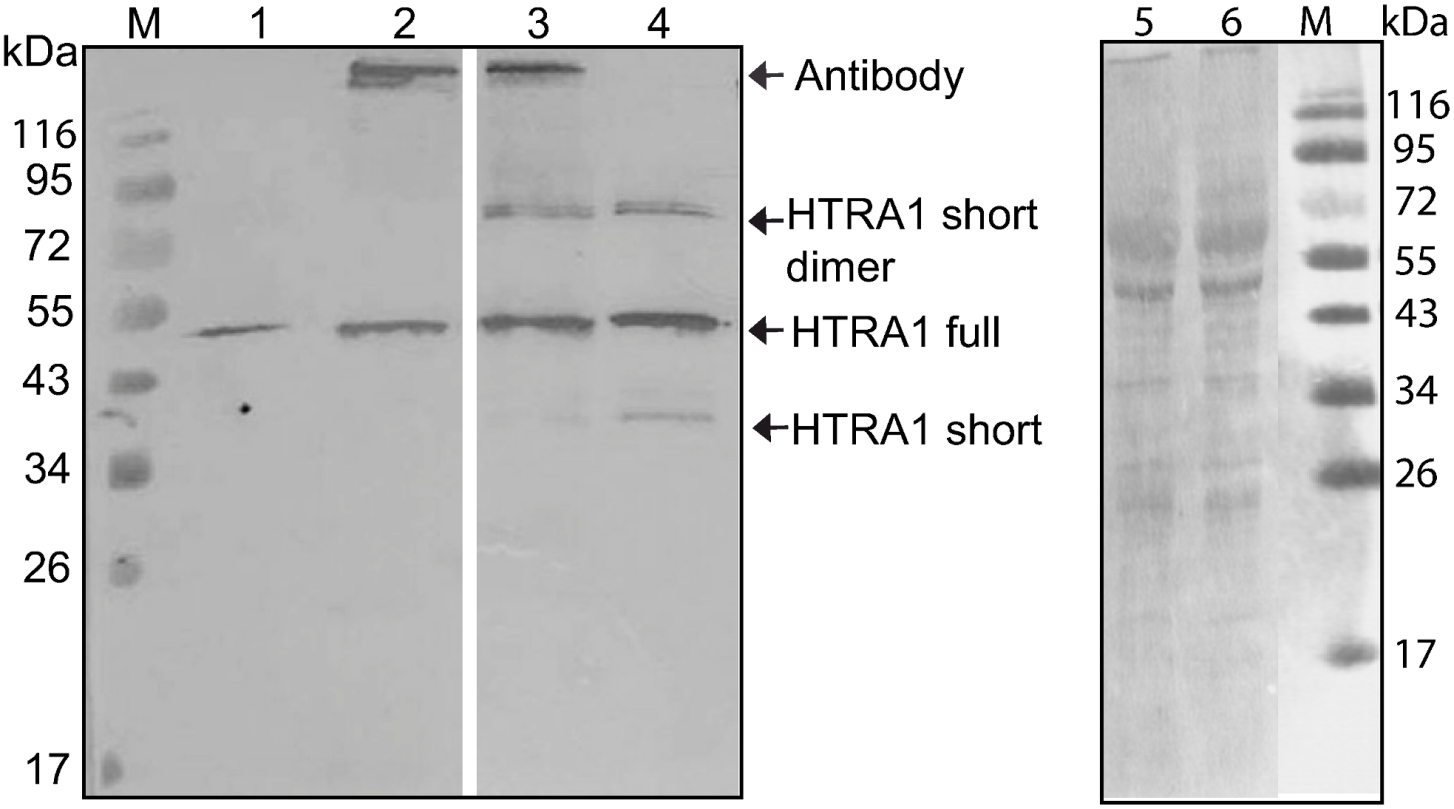
Western Blot analysis of placenta proteins after enrichment using immunobeads. The anti-HTRA1 antibody detected the 50 kDa (HTRA1 full) and the 37 kDa (HTRA1 short) recombinant HTRA1 forms expressed in insect cells (control: lane 1: 50 ng HTRA1 full, lane 4: 200 ng HTRA1 full). Lane 2 shows the placenta proteins after treatment with 10 µl immunobeads. Lane 3: Placenta proteins with 200 ng recombinant HTRA1 full after treatment with 10 µl immunobeads, to verify the binding capacity of the beads. Lane 5 and 6 show the placenta proteins before and after the extraction of HTRA1. Without enrichment, only unspecific signals were found. Based on the control, total amount of HTRA1 in lane 2 was estimated to 150 ng. The image is representative of two independent experiments.

To detect HTRA1 in the placenta protein extract, we enriched HTRA1 from the placenta proteins using immunobeads. The concentration of enriched HTRA1 was then estimated by Western blot using a monoclonal HTRA1 antibody and applying known amount of recombinant HTRA1 expressed in insect cells (Fig. 1). The monoclonal HTRA1 antibody recognizes the full-length HTRA1 at ∼50 kDa, the ∼37 kDa truncated monomer and the ∼74 kDa dimer of recombinant truncated HTRA1. Without enrichment, only unspecific signals were found (Fig. 1 lane 5 and 6). After enrichment, one signal assigned to the full-length HTRA1 appeared on the Western blot (Fig. 1, lane 2 and 3). Approximately 150 ng full-HTRA1 was extracted from 15 mg placenta proteins. The same antibody against HTRA1 was tested for immunohistochemistry on placental tissue with the aim to show the co-localization of protease and candidate substrates, but this antibody was not compatible with formalin fixed tissue.

### Screening for substrates of HTRA1 in placental tissue

**Figure 2:**
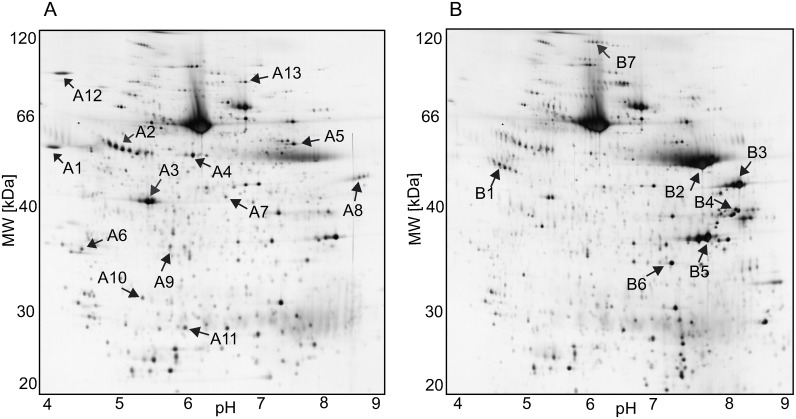
Details of the 2-D patterns of placental proteins incubated with HTRA1. The placenta proteins were incubated for 4 h at 37°C without (A) or with the protease HTRA1 (B). Numbers indicate protein spots that were subjected to tryptic in-gel digestion and LC-MS (A = disappeared spots, B = appeared spots). Details and accession no of the substrates can be found in Table 1. The shown images are representative of four independent experiments, one in the presence of the inhibitor Timp-1 and one in the presence of an inhibitor cocktail (Fig. S2 and S3).

For the identification of the candidate substrates of HTRA1 in the placenta we used a gel-based proteomics approach. The proteins for the screening experiments were extracted from human placental tissue as described in the experimental part. The placenta protein extract was incubated with or without addition of recombinant HTRA1 expressed in insect cells and potential substrates were identified by comparing the protein patterns of 2-DE separation followed by LC-MS analysis of the tryptic in-gel digest. The reduction in spot intensity on the 2-DE pattern on the gel representing the placenta protein extract incubated with HTRA1 compared with the 2-DE patterns of the control indicated intact substrates. The appearance of new spots indicated possible cleavage fragments (Fig. 2 and Fig. S1). Proteins were considered to be a potential substrate when the spot corresponding to the full-length product disappeared or was significantly reduced. The proteins underlying the spots A1 to A13 and B1 to B7 labeled in Figure 2 were chosen as potential substrates. These spots were cut, removed and the proteins were digested in-gel using trypsin. The obtained peptides were analysed by LC-MS and identified with protein data base with a search engine. With this approach we identified several potential substrates (Table 1, Fig. 2, for more details see Table S1). Among the potential substrates, proteins of the cytoskeleton, proteins of the disulfide-isomerase family, several heat shock proteins, some proteins responsible for the protein biosynthesis and the protease inhibitor alpha-1-antitrypsin were identified (Table 1). The Spots B2–B6 corresponded to the added protease HTRA1 and to HTRA1 fragments. The degradation of HTRA1 after several hours of incubation at 37°C in the presence of other proteins (substrates or non-substrates) or in PBS buffer was regularly observed in this study and is probably an autocatalytic process.

**Table 1:** HTRA1 candidate substrates identified in the placenta protein.

Spot number	Protein name	Name	M [kDa]	localization	Accession number
A1	Protein disulfide-isomerase	PDIA1	57	Membrane, ER	P07237
A2, B1	Alpha-1-antitrypsin	A1AT	46	Secreted, ECM	P01009
A3	Actin cytoplasmic 1	ACTB	41	Cytoplasm,	P60709
	Actin, aortic smooth muscle	ACTA	42	cytoskeleton	P62736
A4	Protein disulfide-isomerase A3	PDIA3	56	ER	P30101
A5	Pyruvate kinase isozymes M1/M2	KPYM	58	Cytoplasm,nucleus	P14618
A6	Tropomyosin β-chain	TPM2	32	Cytoplasm,cytoskeleton	P07951
A7	Adenosyl homocysteinase	SAHH	47	Cytoplasm	P23526
A8	Elongation Factor –α 1	EF1A1	50	Cytoplasm,nucleus	P68104
A9	Estradiol 17-β-dehydrogenase 1	DHB1	35	Cytoplasm	P14061
A10	Chloride intracellular channelprotein 1	CLIC1	26	Membrane,cytoplasm,nucleus	O00299
A11	Heat shock protein β-1	HSPB1	23	Cytoplasm,cytoskeleton	P04792
A12	Endoplasmin	ENPL	92	ER	P14625
A13	Elongation factor 2	EF2	95	Cytoplasm	P13639

ECM: extracellular matrix. 2D_A3: ACTB and ACTA are proteins of the actin family with very similar sequences.

### Investigation of the proteolytic activity of HTRA1 in the presence of inhibitors

Many proteases are part of proteolytic cascades. If HTRA1 is integrated in such a cascade it may activate other proteases. These activated proteases will then digest further substrates which are not substrates of HTRA1 but could be misinterpreted as HTRA1 substrates. The identification of A1AT as a candidate substrate of HTRA1 raises the idea that the proteolytic activity of proteases will increase which are inhibited by A1AT, if the concentration of the latter decreases. To reduce the probability that other proteases than HTRA1 are responsible for degrading placenta tissue proteins, we looked for protease inhibitors which do not inhibit HTRA1. Therefore β-casein, a known substrate of HTRA1, was incubated with HTRA1 in the presence and in the absence of several inhibitors.

**Figure 3:**
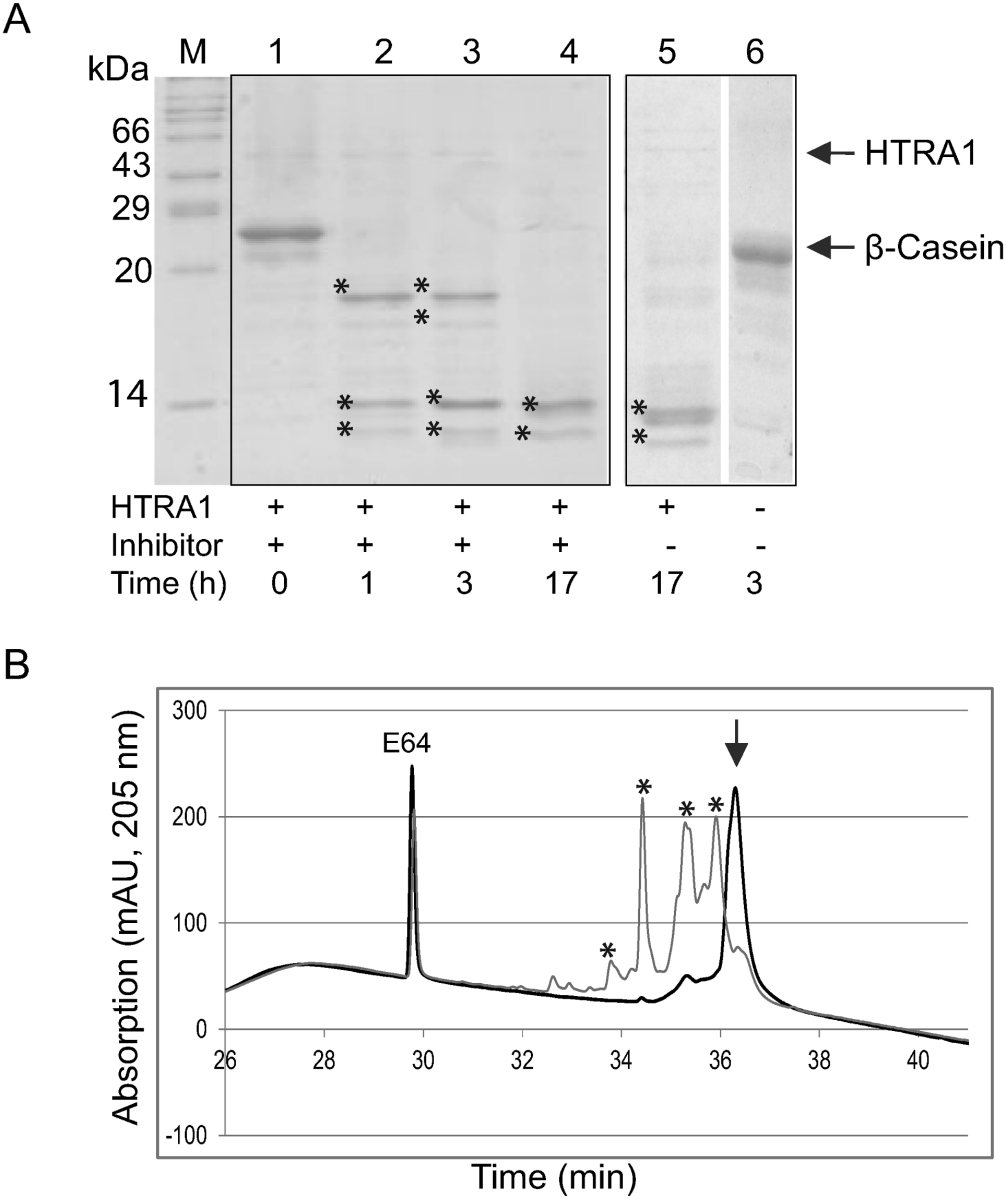
Influence of protease inhibitors on proteolytic activity of HTRA1, exemplified with E64. A: SDS-PAGE separation of the incubation products of HTRA1 and β-casein in the presence of E64. Lanes 1 to 4: 0 h, 1 h, 3 h and 17 h. Lane 5: incubation of HTRA1 and β-casein without E64, 17 h. Lane 6: incubation of β-casein without HTRA1 and without inhibitor for h. The proteolytic activity of HTRA1 in the presence of E64 is 100% compared to the control. The arrows indicate HTRA1 (above) and β-casein; the asterisks indicate the cleavage products of β-casein. M, marker. B: The proteolytic activity in the presence of E64 was monitored by HPLC analysis using UV detection. Incubation time 0 h (thick line) and 1 h (thin line). Height of the β-casein peak was used for relative quantification (mean of three independent replicates, Table 2). The asterisks indicate the cleavage products of β-casein. The arrow indicates β-casein. MS analysis was used to verify the identity of the β-casein peak (36.3 min).

The protease activity of HTRA1 was not significantly inhibited by the serine protease inhibitors aprotinin, and AEBSF, the cysteine protease inhibitors leupeptin and E64, the metalloprotease inhibitor bestatin and the aspartic protease inhibitor pepstatin A (Fig. 3, Table 2). The chelator EDTA slowed slightly the proteolytic reaction, leading to the presumption, that metal ions like Ca^2+^ have a supporting effect on the catalysis of HTRA1 although HTRA1 is not a metalloprotease (Table 2).

**Table 2:** Influence of different protease inhibitors on the proteolytic activity of HTRA1.

Inhibitor	Concentration	β-casein after 1 h [%]
Control	-	21+/−4
EDTA	10 mM	33+/−5
E64	10 µM	25+/−2
Pepstatin A	700 mM	18+/−3
Aprotinin	800 nM	23+/−2
Bestatin	40 µM	25+/−3
Leupeptin	50 µM	23+/−2
AEBSF	0.2 mM	24+/−5

The protease inhibitors were added separately to an incubation mixture containing 5 µg β-casein and 0.5 µg HTRA1 at 37°C. Aliquots were taken at time 0 h and after 1 h incubation at 37°C and analysed by RP-HPLC. Percentage degradation of β-casein was calculated on the height basis from the UV chromatogram obtained at a wavelength of 205 nm, normalised to the height of the β-casein peak at time 0 h (mean of three replicates). As control, β-casein was incubated with HTRA1 without addition of an inhibitor. Compared with the control, protease activity of HTRA1 was not significantly inhibited by the inhibitors tested in this study. Only EDTA seems to slow slightly the proteolytic reaction.

Based on these results, placenta proteins were incubated with or without HTRA1 (control sample) for 4 hours at 37°C in the presence of a protease inhibitor cocktail (1% of the reaction volume) which contained AEBSF, E64, aprotinin, bestatin hydrochloride, leupeptin hemisulfate salt, and pepstatin A. Compared with the results of the first 2-DE analysis described above, the presence of the protease inhibitor cocktail did almost not change the 2-DE gel pattern of the protease treated sample and the same candidate substrates were identified (Fig. S2).

As the candidate substrate A1AT is known to be a substrate of several MMPs [28], [29], [30], we repeated the incubation experiment of placenta proteins in the presence or absence of HtrA1 and in presence of the MMP inhibitor Timp-1. Here again, there was no significant change in the 2-DE gel pattern compared to the first 2-DE data describe above (Fig. S3).

### A1AT as target of HTRA1

**Figure 4:**
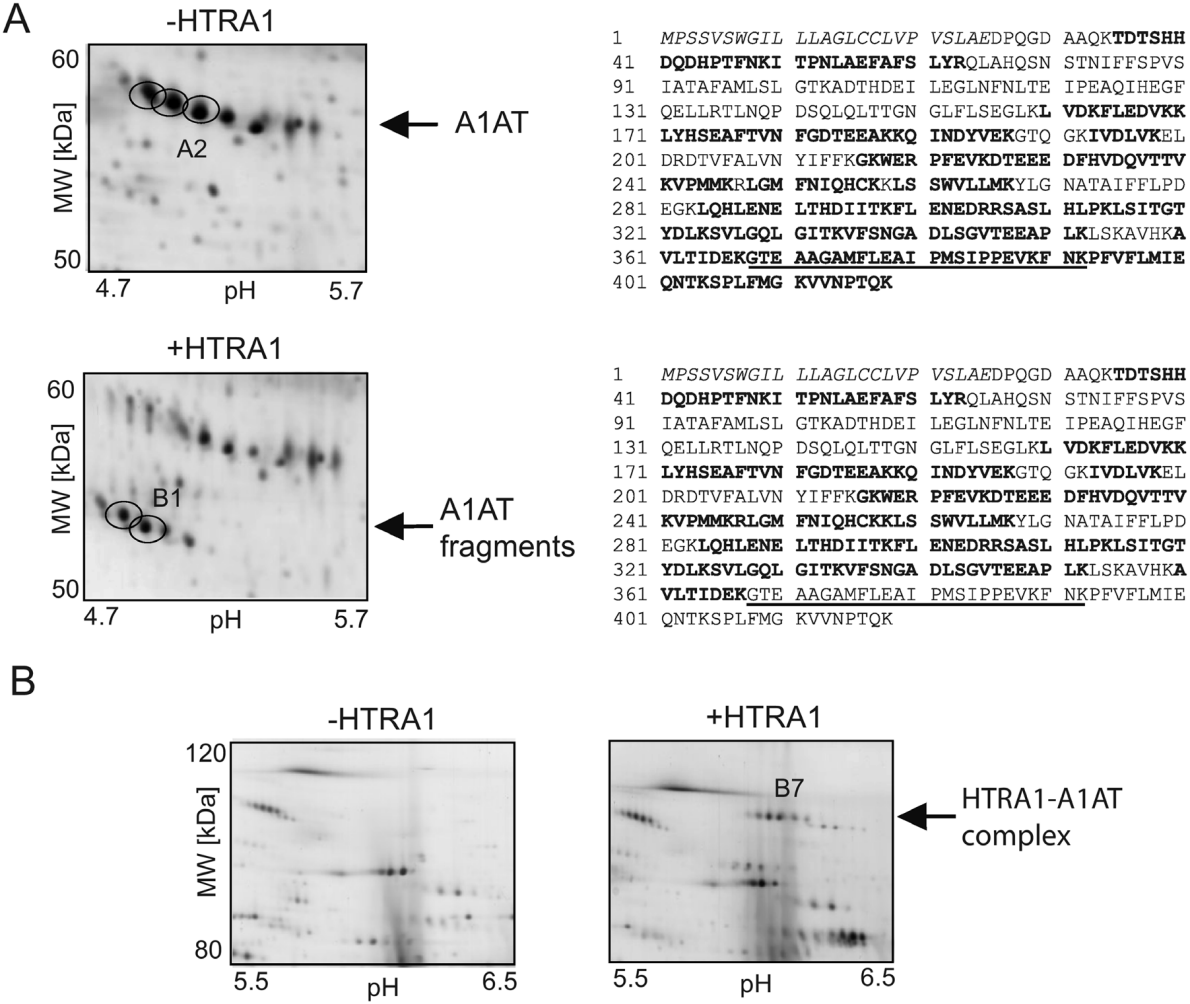
Details of the 2-D patterns of the candidate substrate A1AT incubated with HTRA1. Details of the 2-D pattern of the HTRA1 substrate A1AT in the placenta protein sample after incubation in the absence (−HTRA1) and in the presence (+HTRA1) of HTRA1 (A). The images are small sections of the Figure 2. The arrows indicate placenta A1AT (spots A2) and placenta A1AT fragments (Spots B1). On the right hand site: Sequences of A1AT identified by MS analysis (bold sequence) after tryptic in-gel digestion of the spots A2 and B1. The italic sequence indicates the signal peptide; the underlined sequence indicates the reaction center loop. (B) A1AT forms a stable complex with HTRA1 at high molecular weight range (spot B7); this was supported by MS measurements. Three replicates are shown in Figure S6.

Among the candidate substrates identified, A1AT was chosen for further validation experiments. A1AT was considered to be of particular interest because of its important role as protease inhibitor. A1AT is a high abundant plasma protein and is also expressed in the placenta in high concentrations [31]. Interestingly, A1AT is able to form a stable complex with HTRA1. This behaviour previously reported by Hu et al. [4], was verified in our study. On the 2-DE of the incubated sample of the placenta proteins and HTRA1, spots shifted from 55 kDa (A1AT) to approximately 120 kDa indicating the presence of the A1AT-HtrA1 complex (Fig. 4). This was confirmed by LC-MS analysis of the spot B7 after tryptic in-gel digestion.

A1AT is a highly glycosylated protein (asparagine 46, 83 and 247). Heterogeneity of the glycans (bi- and tri-antennary) gives rise to multiple protein species [32]. On the 2-DE several spots with pI shift of nearly 0.1 units and mass decrease of approximately 2 kDa are typical for A1AT [32] (Fig. 4, Fig. S6). The HTRA1 generated A1AT fragments show very similar species pattern with a decrease in the molecular weight of nearly 4 kDa compared to the full-length A1AT species. This similar pattern suggests that the glycosylation sites do not have an impact on the proteolytic action of HTRA1. Furthermore it can be assumed that HTRA1 cleaves the different A1AT species at one specific cleavage site.

To get more information about the cleavage site of HTRA1 in A1AT, the full A1AT and the fragment spots were analysed with MS, after trypsin digestion and LC separation. Compared with the full A1AT, peptides from the C-terminus were not detected in the fragment spots (Fig. 4). These results suggest a cleavage site within the C-terminal part of A1AT.

**Figure 5:**
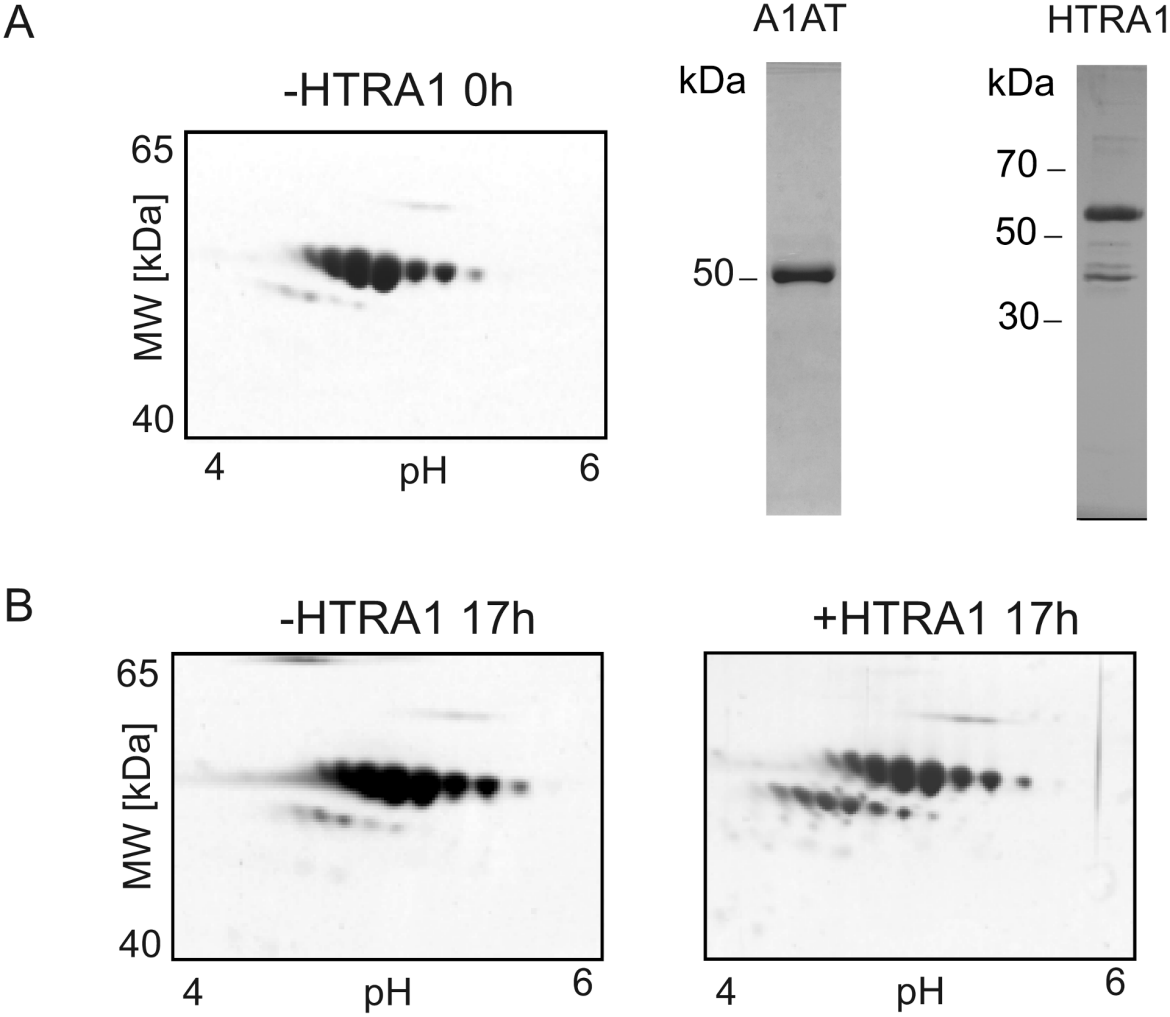
Validation of the candidate substrate A1AT of HTRA1. Purified A1AT from human plasma was incubated with HTRA1 at 37°C for 17 hours. (A) 2-D gel of A1AT in the absence of HTRA1 incubation time 0 h and SDS-PAGE of A1AT and HTRA1. The A1AT preparation contains only very small amount of cleaved A1AT. (B) Incubation over a period of 17 hours in the absence (left site) and presence (right site) of HTRA1. After incubation with HTRA1 a significant amount of A1AT was cleaved. The incubation of plasma A1AT with HTRA1 showed similar fragmentation patterns as the incubation of placental A1AT with HTRA1 (Fig. 4A).

To validate the degradation of A1AT by HTRA1, purified A1AT from human plasma (ProteaImmun) was incubated with the protease in a ratio of protease to substrate of 1∶10. The incubated sample and the control samples (time 0 h and overnight of incubation without HTRA1) were separated using 2-DE. Incubation generated cleavage products with a mass difference of nearly 4 kDa (Fig. 5). The incubation samples were also subjected to SDS-PAGE (Fig. S4). Selected protein spots of the SDS-PAGE were in-gel digested with trypsin and analysed by MS (spots 1D_A1, 1D_A2 and 1D_A3). Peptides of the C-terminal part of A1AT could be detected in the spot 1D_A1 but not in the spot 1D_A3 (cleavage product). A1AT and HTRA1 were both identified in spot 1D_A2. These results confirm that in the cleavage products of A1AT the C-terminal part is missing and that HTRA1 and A1AT are able to build a stable complex.

The protein A1AT is an inhibitor for a range of serine proteases. As HtrA1 is a serine protease, we tested if the presence of A1AT is affecting the activity of HTRA1. Therefore A1AT and HTRA1 were incubated in a ratio 50∶1 followed by addition of the substrate β-Casein. The proteolytic activity of HTRA1 was monitored using SDS Page of aliquots of the incubation mixture taken at different times after the addition of β-Casein. Proteolysis of β-casein through the protease HtrA1 still takes place after 1 hour incubation. No significant difference was observed compared to the sample without A1AT (Fig. S5).

### Mass spectrometric cleavage-site identification of HtrA1 in A1AT

**Figure 6:**
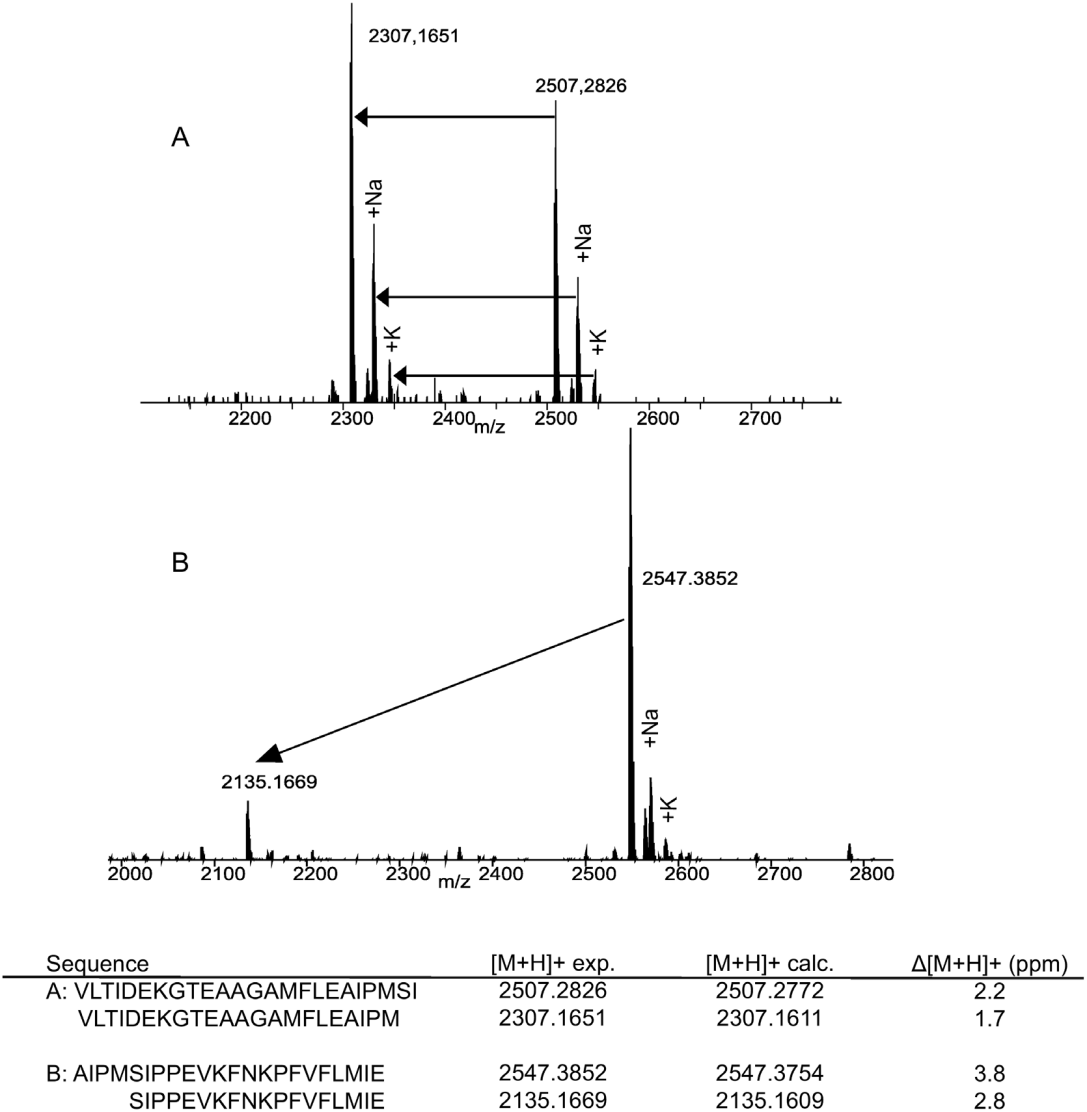
MALDI-MS spectra after incubation of A1AT peptides with HTRA1. MALDI-Orbitrap-MS analysis as described in experimental procedures. A: Peptide A1AT(361–384) and generated fragment. Cleavage occurs after met381. B: Peptide A1AT(379–400) and generated fragment. Cleavage occurs again after met381. Further signals are sodium adduct (+22) and potassium adduct (+38). The image is representative of three independents experiments. The same incubation experiment was performed without addition of HTRA1 as negative control (Fig. S7).

To detect the exact cleavage site of HTRA1 in A1AT, three different peptides were synthetized. The peptides contained sequence part of A1AT where the cleavage site is supposed to be, according to the MS analysis of the A1AT-fragment spots of the 2-DE experiments. The synthetic peptides were: A1AT(361–384), A1AT(379–400) and A1AT(396–418). They were incubated each separately with HTRA1 in a molar ratio of protease to peptide of 1∶10 at 37°C over night, and the proteolytic products were identified by MALDI-Orbitrap MS (Fig. 6). Incubation of the A1AT peptides without HTRA1 (negative control) generated no cleavage fragments (Fig. S7). Incubation of the peptide A1AT(361–384) with HTRA1 generated the fragment (361–382), thus cleavage occurred after met-382. The same specific cleavage site was found after incubation of the peptide A1AT(379–400) with the protease, as the fragment (383–400) was generated. Incubation of A1AT(396–418) produced as expected no cleavage fragments (Fig. S8).

## Discussion

In this work the total amount of HTRA1 in human placenta tissue was estimated to be in a 10^−5 ^mg range per 1 mg total protein using a western blot. This corresponds to nearly 0.001% of the protein in the placenta. Interestingly, only the full-length form of HTRA1 was detected on the western blot, although the monoclonal HTRA1 antibody is also able to recognise the truncated HTRA1 form. This result coincides to findings of Lorenzi et al. [21]. They describe a high amount of the full-length form in healthy human placenta and, in contrast to that, a total down-regulation of the full-length form and an up-regulation of a 30 kDa truncated form of HTRA1 in preeclampsia placenta. The authors suggested that the truncated HTRA1 form has a physiological relevance and may be responsible for maldevelopment of the villi through degradation of fetal vessel fibronectin in placental villi.

High levels of HTRA1 were found in the third trimester of pregnancy compared to the first and second trimester [19]. HTRA1 is localized in the cytoplasm of the placenta cells and in the extracytoplasmic space of the stroma of placenta villi. In the first part of pregnancy HTRA1 is expressed in the syncytiotrophoblast and in the cytotrophoblast, the both layers surrounding placenta villi. In the third trimester, the expression is higher in the syncytiotrophoblast, the cell layer attending to maternal blood. The syncytiotrophoblast layer is continuously regenerated during pregnancy by fusion of the underlying trophoblast cells, as its translation rate is low and the cells non-proliferative [33]–[35]. It is responsible for the transfer of oxygen and nutrients between the maternal blood and the fetus. Moreover, it is the place where many hormones required for fetal growth are synthetized. One of the identified potential substrates for HTRA1 in this study is the protease inhibitor alpha-1-antitrypsin (A1AT). The localization pattern of A1AT in the placenta shows similarity with the localization pattern of HTRA1. Both are located in the syncytiotrophoblast in mature placenta [19] [31]. Results of the Human Protein Atlas Project, publicly available online (www.proteinatlas.org) also show that HTRA1 and A1AT are both located in the trophoblastic cells of the placenta [36], [37]. This is important, as we used a homogenate of placental tissue containing proteins of different cell-types. The correlation of the localization pattern supports the assumption, that A1AT is a biological relevant substrate of HTRA1. As A1AT is a protease inhibitor, its inactivation through cleavage may have important consequences for the protease and protease inhibitor balance of the cell. Thus, A1AT was considered to be of particular interest and was chosen for further validation experiments.

Interestingly, incubation of placental A1AT with HTRA1 led to a limited proteolysis of A1AT. After cleavage, a decrease in molecular weight of nearly 4 kDa was observed. Similar results were obtained after incubation of purified A1AT from human plasma with HTRA1. A specific cleavage site was identified after met-382 (AIPM^382^↓^383^SIPP) and the generated fragment is the residue C-36. The C-terminus of A1AT is crucial as it contains the reactive centre loop RCL (AA 368–392) which mediates the protease inhibitory property by binding to the target. The c-terminal part of A1AT seems to be particularly susceptible to proteolysis by a number of proteases ([38], MEROPS database, http://merops.sanger.ac.uk/index.shtml). For example matrix metallopeptidase-11 cleaves after ala-374 [28], thermolysin after met-375 [39] and periodontain after glu-378 [40]. The cleavage site after met-382 was reported for proteases such, as cathepsin L [41], elastase-2 [40] and chymotrypsin A [39]. Johnson et al. show that cleavage of A1AT after met-382 leads to the inactivation of the protease inhibitor. Thus, the consequence of the cleavage of A1AT through HTRA1 will in a similar way leads to an inactivation of the protease inhibitor.

A1AT is a suicide protease-inhibitor with an extraordinary inhibitor mechanism. First, A1AT binds to the protease through residue of the RCL part. Then the protease cuts the protease inhibitor and a covalent acyl bond is built between A1AT and the protease. In response to the formation of this covalent bond A1AT changes its conformation causing a deformation of the protease, thereby inactivating it through formation of a very stable complex [42]. For instance the complex A1AT-chymotrypsin dissociates only after three to four days [43]. Finally, the covalent complex can be bound by receptors and degraded in lysosomes [44]. In contrast, the reaction of placental A1AT with HTRA1 leads not only to the formation of a protease-protease inhibitor complex, but also to the formation of cleavage fragments of A1AT within 4 hours. This leads to the assumption that either the A1AT-HTRA1 complex is not as stable as other A1AT-protease complexes, and dissociates after less than 4 hours into protease and the cleaved inactive protease inhibitor, or the reaction mechanism is a branched one, as already suggested by Patston et al. [45] for other protease inhibitors of the serpin family to which A1AT belongs [45]–[47]. It is challenging to predict how the reaction is regulated in the biological context and if A1AT is acting as a substrate or rather as an inhibitor for the protease HTRA1. Furthermore, the high concentration of HTRA1 used in our experiments could influence the affinity of protease and protease-inhibitor in an artificial way. This remains to be further investigated in the future in a cellular context. Nevertheless, the consequences are the cleavage and inactivation of the protease inhibitor A1AT and this may have important consequences [48], [49]. A disorder of the protease and protease inhibitor balance may be associated with pathophysiological events. Cleaved A1AT is also known to polymerize by β-strand linkage [50]. Accumulation of A1AT polymers can lead to severe diseases [51]. As the syncytiotrophoblast layer is directly attending to maternal blood, the inactivation of A1AT may have an impact on blood pressure regulation. The level of active proteases which are inhibited by A1AT could increase, as for instance neutrophil elastase, a protease participating in inflammation and responsible for reduced blood pressure through degradation of elastin in the arterial wall [49].

It was previously reported, that HTRA1 could be involved in apoptotic process, through degradation of proteins of the cytoskeleton as tubulin and microtubules, and of the apoptosis inhibitor XIAP [14], [15] and to promote anoikis [52]. Since the protease inhibitor A1AT was reported to have an anti-apoptotic property possibly due to interaction with caspase-3 [53], the apoptosis supporting effect of HTRA1 may be also induced by diminishing the inhibitory property of A1AT.

The syncytiotrophoblast layer is formed from continuous fusion of the cytiotrophoblast. Most of the differentiated trophoblast cells are required to transport mRNA to the syncytium rather than for its growth [35]. Excessive syncytial formation is extruded in maternal blood as syncytial knots (accumulated apoptotic nuclei). This continuous turnover has to be tightly regulated. We suggest that HtrA1 may be involved in the regulation of apoptotic processes of the syncytiotrophoblast through inactivation of A1AT.

In this work, we have also identified two further members of the cytoskeleton, actin cytoplasmic 1 and tropomyosin beta as candidate substrates of HTRA1. Both are involved in cell motility and stability. Degradation of actin cytoplasmic 1 and tropomyosin beta by HTRA1 could play a role in cytoskeletal remodelling during apoptotic process, similar to the degradation of tubulin and microtubules.

Other candidate substrates identified here suggest that HTRA1 may be involved in protein biosynthesis process (EEF2, EEF1A1) and protein folding (HSPB1, PDIA1, PDIA3, HSP90B1), however, the significance of their degradation needs further investigation.

In conclusion, this study revealed several new candidate substrates of the protease HTRA1, which provides an insight into the function of HTRA1 in the placenta. Alpha-1-antitrypsin, actin cytoplasmic 1, tropomyosin beta chain and ten other proteins were identified as being degraded in the placenta protein sample after incubation with HtrA1. Furthermore we demonstrated that HTRA1 degrades purified plasma A1AT in vitro and, using synthetic peptides covering parts of the sequence of A1AT, we were able to identify a cleavage site within the reactive loop centre of the protease inhibitor. We postulate that HTRA1 may support trophoblast apoptosis, which is essential for the regenerating of the syncytiotrophoblast in the mature placenta and therefore for normal placentation through inactivation of the protease inhibitor A1AT. Main focus of our study was the investigation of potential substrates of HTRA1 in the protein repertoire of the placenta using a screening approach. However, in vivo evidence of the potential substrates identified in this study remains to be verified in cell experiments or animal models. Further studies are needed to confirm the relationship of HTRA1 and A1AT in vivo in the complex context of placenta development and growth.

## Supporting Information

Figure S1 Colour coded picture of the 2-D gel electrophoresis separation of the placenta proteins. Colour coded picture of the two-dimensional protein separation of placenta proteins after 4 h incubation without (blue) and with the protease HTRA1 (orange). This picture was generated by overlaying the two 2-D gels presented in Figure 2 and by using artificial colours. Incubation time 4 hours.
(TIF)

Figure S2 Details of the 2-D patterns of placental proteins incubated with HTRA1 in the presence of the inhibitor cocktail P8340. The placenta proteins were incubated for 4 h at 37°C without (A) or with the protease HTRA1 (B) in the presence of the inhibitor cocktail P8340. The protein endoplasmin was not detected on both 2-DGE. Numbers (A = disappeared spots, B = appeared spots). Details and accession no of the substrates can be found in Table 1.
(TIF)

Figure S3 Details of the 2-D patterns of placental proteins incubated with HTRA1 in the presence of the inhibitor Timp-1. The placenta proteins were incubated 4 h at 37°C without (A) or with the protease HTRA1 (B) in the presence of the inhibitor Timp-1. Numbers (A = disappeared spots, B = appeared spots). Details and accession no of the substrates can be found in Table 1.
(TIF)

Figure S4 SDS-PAGE separation of the incubation products of A1AT with HTRA1. Incubation of A1AT in the absence (A) and in the presence of HTRA1 (B) in a ratio 1∶10. Incubation time: 0 h and overnight. The arrows indicate A1AT, the A1AT-HTRA1 complex and the cleavage products of A1AT. This was confirmed by MS-analysis (Table S1, spots 1D_A1, 1D_A2 and 1D_A3). M: marker, ON: overnight.
(TIF)

Figure S5 HTRA1 is not inhibited through A1AT. SDS-PAGE separation of the incubation products of HTRA1 and β-casein in the absence (A) and in the presence of A1AT (B) in a ration 1∶50∶50 after 0 h, 1 h and 2 h incubation time. The arrows indicate A1AT, HTRA1 and β-casein; the asterisks indicate the cleavage product of β-casein. M: marker. The image is representative of three independent replicates.
(TIF)

Figure S6 Details of the 2-D patterns of the candidate substrate A1AT incubated with HTRA1 three replicates shown. The placenta proteins were incubated 4 h at 37°C without (negative control Fig. A and C) or with the protease HTRA1 (Fig. B and D). A1AT forms a stable complex with HTRA1 at high molecular weight range (Fig. B, spot B7). No complex is observed when the placenta proteins are incubated without HTRA1 (A). A1AT species are significantly digested by HTRA1, new spot formations on the protease-treated sample are fragments of A1AT (Fig. D, spots B1). Control sample shows the A1AT species after incubation without HTRA1 (Fig. C). Details pictures lanes 1 and 2: two independent experiments. Details pictures lane 3: independent experiment in the presence of the inhibitor Timp-1.
(TIF)

Figure S7 MALDI-MS spectra after incubation of A1AT peptides without HTRA1 (negative control). MALDI Orbitrap-MS analysis as described in experimental procedures. Spectra of the peptides after incubation without HTRA1. A: Peptide A1AT(361–384) B: Peptide A1AT(379–400).
(TIF)

Figure S8 MALDI-MS spectra after incubation the peptide A1AT(396–418) with HTRA1. MALDI Orbitrap-MS analysis as described in experimental procedures. A: Peptide A1AT(396–418) after incubation with HtrA1. No cleavage fragment was generated. B: Peptide A1AT(396–418) after incubation without HTRA1 (negative control).
(TIF)

Table S1 List of candidate substrates of HTRA1 identified in the placenta. Spot 2D_A3: nearly same peptides were identified for the protein P60709 and P62736 (actin, aortic smooth muscle). 2D_A2 and 2D_A5: the protein P01019 (angiotensinogen) and P01857 (Ig gamma-1 chain C region) were also identified and stem probably from low abundant underlying spots. 2D_B7: the protein serum albumin stems from the huge protein spot at mass 66 kDa (Fig. 2). In spot 1D_A2 there is no serum albumin contamination. A1AT*: cleaved A1AT.
(DOCX)
