# Supplementary figures and images for: Alpha-1-Antitrypsin: A Novel Human High Temperature Requirement Protease A1 (HTRA1) Substrate in Human Placental Tissue
Source: PLoS One. 2014 Oct 20;9(10):e109483. doi: 10.1371/journal.pone.0109483 (PMC4203740; doi:10.1371/journal.pone.0109483)

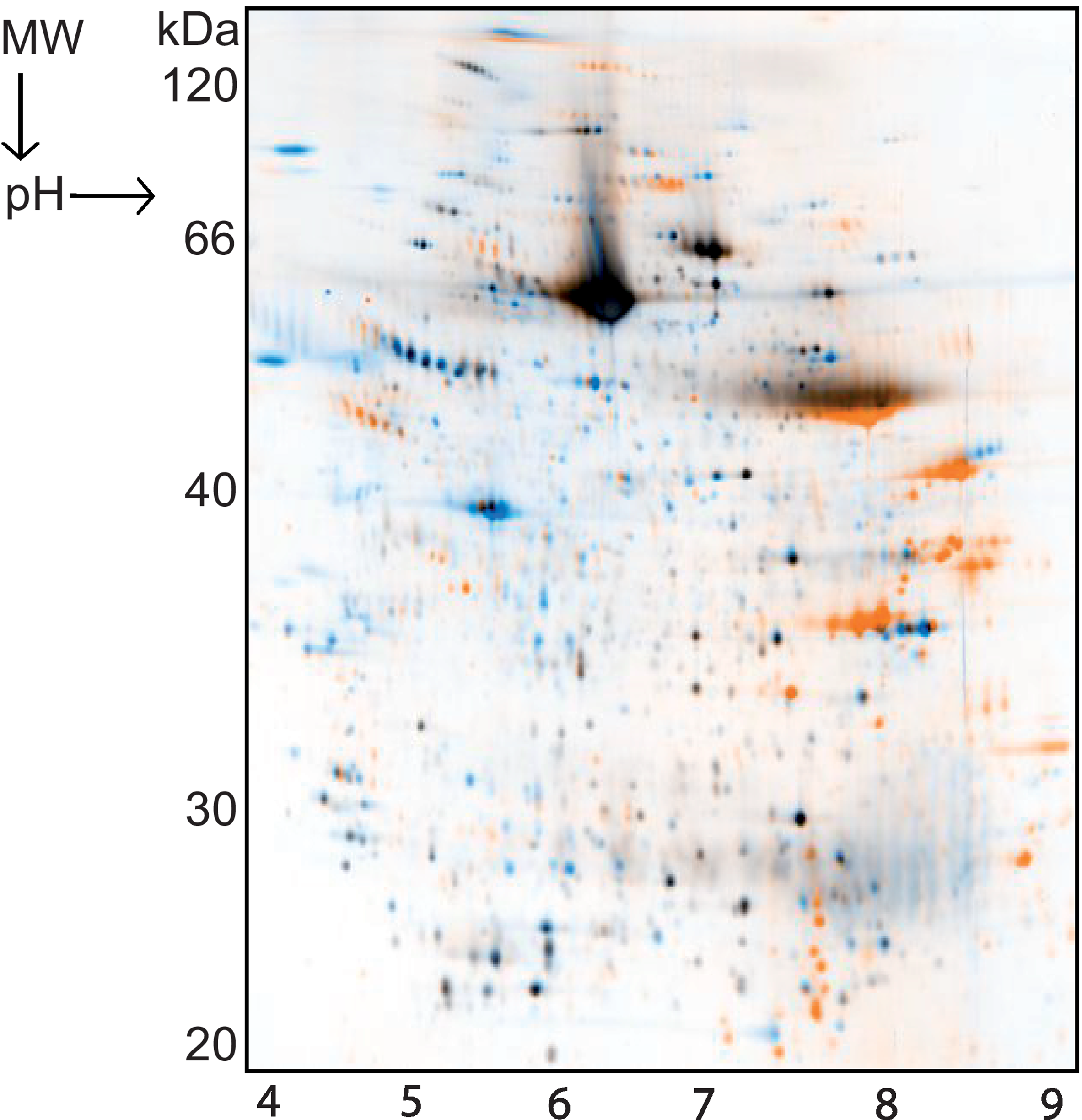

Supplement: Figure S1 — Colour coded picture of the 2-D gel electrophoresis separation of the placenta proteins. Colour coded picture of the two-dimensional protein separation of placenta proteins after 4 h incubation without (blue) and with the protease HTRA1 (orange). This picture was generated by overlaying the two 2-D gels presented in Figure 2 and by using artificial colours. Incubation time 4 hours. (TIF) [file pone.0109483.s001.tif]
