# Supplementary material for: Alpha-1-Antitrypsin: A Novel Human High Temperature Requirement Protease A1 (HTRA1) Substrate in Human Placental Tissue
Source: PLoS One. 2014 Oct 20;9(10):e109483. doi: 10.1371/journal.pone.0109483 (PMC4203740; doi:10.1371/journal.pone.0109483)
Supplement: Table S1 — List of candidate substrates of HTRA1 identified in the placenta. Spot 2D_A3: nearly same peptides were identified for the protein P60709 and P62736 (actin, aortic smooth muscle). 2D_A2 and 2D_A5: the protein P01019 (angiotensinogen) and P01857 (Ig gamma-1 chain C region) were also identified and stem probably from low abundant underlying spots. 2D_B7: the protein serum albumin stems from the huge protein spot at mass 66 kDa (Fig. 2). In spot 1D_A2 there is no serum albumin contamination. A1AT*: cleaved A1AT. (DOCX) [file pone.0109483.s009.docx]

**Table S1.** List of candidate substrates of HTRA1 identified in the placenta. Spot 2D_A3: nearly same peptides were identified for the protein P60709 and P62736 (actin, aortic smooth muscle). 2D_A2 and 2D_A5: the protein P01019 (angiotensinogen) and P01857 (Ig gamma-1 chain C region) were also identified and stem probably from low abundant underlying spots. 2D_B7: the protein serum albumin stems from the huge protein spot at mass 66 kDa (Figure 2). In spot 1D_A2 there is no serum albumin contamination. A1AT*: cleaved A1AT.

| **Spots** | **Accession numbers** | **Name** | **unique Peptide** | **Sequence coverage %** | **Score**  **SEQUEST** |
| --- | --- | --- | --- | --- | --- |
| 2D_A1 | P07237 | PDIA1 | 22 | 68 | 499 |
| 2D_A2 | P01009  P01019 | **A1AT**  **ANGT** | 23  8 | 66  24 | 573  78 |
| 2D_A3 | P60709  P62736 | ACTB  ACTA | 3  3 | 38  42 | 357  293 |
| 2D_A4 | P30101 | PDIA3 | 28 | 69 | 578 |
| 2D_A5 | P14618  P01857 | KPYM  IGHG1 | 23  3 | 71  37 | 516  53 |
| 2D_A6 | P07951 | TPM2 | 9 | 50 | 182 |
| 2D_A7 | P23526 | SAHH | 5 | 25 | 54 |
| 2D_A8 | P68104 | EF1A1 | 4 | 16 | 47 |
| 2D_A9 | P14061 | DHB1 | 7 | 38 | 127 |
| 2D_A10 | O00299 | CLIC1 | 5 | 46 | 81 |
| 2D_A11 | P04792 | HSPB1 | 3 | 40 | 80 |
| 2D_A12 | P14625 | ENPL | 26 | 38 | 382 |
| 2D_A13 | P13639 | EF2 | 10 | 22 | 134 |
| 2D_B1 | P01009 | A1AT* | 11 | 47 | 104 |
| 2D_B2 | Q92743 | HTRA1 | 15 | 50 | 251 |
| 2D_B3 | Q9274 | HTRA1 | 12 | 41 | 233 |
| 2D_B4 | Q9274 | HTRA1 | 6 | 31 | 91 |
| 2D_B5 | Q9274 | HTRA1 | 10 | 55 | 92 |
| 2D_B6 | Q9274 | HTRA1 | 4 | 22 | 75 |
| 2D_B7 | P01009  Q9274  P02768 | HtrA1  A1AT  ALBU | 5  5  6 | 23  22  18 | 89  73  55 |
| 1D_A1 | P01009 | A1AT | 12 | 39 | 477 |
| 1D_A2 | P01009  Q9274 | A1AT  HTRA1 | 21  14 | 63  50 | 382  309 |
| 1D_A3 | P01009 | A1AT* | 37 | 65 | 128 |
